# The Biomarkers for Identifying Preclinical Alzheimer's Disease via Structural and Functional Magnetic Resonance Imaging

**DOI:** 10.3389/fnagi.2016.00092

**Published:** 2016-04-27

**Authors:** Yanhong Zhou, Chuangchuang Tan, Dong Wen, Hongmin Sun, Wei Han, Yuchen Xu

**Affiliations:** ^1^Department of Computer Science and Technology, School of Mathematics and Information Science & Technology, Hebei Normal University of Science and TechnologyQinhuangdao, China; ^2^Department of Electronic Information Science and Technology, School of Sciences, Yanshan UniversityQinhuangdao, China; ^3^Department of Computer Science and Technology, School of Information Science and Engineering, Yanshan UniversityQinhuangdao, China; ^4^The Key Laboratory for Computer Virtual Technology and System Integration of Hebei Province, Yanshan UniversityQinhuangdao, China; ^5^Department of Physical Education, School of Physical Education, Yanshan UniversityQinhuangdao, China

**Keywords:** preclinical Alzheimer's disease, structural magnetic resonance imaging, functional magnetic resonance imaging, mild cognitive impairment, gene, protein, cognitive reserve

## Introduction

Preclinical Alzheimer's disease (Pre-AD) has been regarded as the preclinical pathological state of mild cognitive impairment (MCI) and Alzheimer's disease (AD; Sperling et al., 2011), and becomes a popular research field currently. At present, many researchers studied the biomarkers of Pre-AD from several angles, including of neuropsychology scales (Duara et al., 2011), structural Magnetic Resonance Imaging (sMRI; Wang et al., 2013), functional Magnetic Resonance Imaging (fMRI; Brier et al., 2014), Positron Emission Tomography (Doraiswamy et al., 2012), cerebrospinal fluid (Moghekar et al., 2013), gene (Ferencz et al., 2012), and cognitive reserve (Soldan et al., 2015), and then made a series of achievement. Considering of simpleness and shortcut of diagnosis, sMRI, and fMRI as important biomarkers become the major inspection means of cerebral disease in clinical tests increasingly. At present, many studies analyzed the biomarkers of Pre-AD in depth, including of cerebral structure, brain functional connection, brain networks and their relationship with gene and protein of Pre-AD from sMRI, and fMRI aspects (Brier et al., 2014; Besson et al., 2015; Gordon et al., 2015; Miller et al., 2015; Harrison et al., 2016). However, these studies are still in early stages, and many conclusions need to be further verified. In addition, the data processing and analysis methods in these studies remain to be further explored, and there still exist many unsolved problems.

Therefore, this paper will analyze in depth the literatures related to Pre-AD from the angles of sMRI and fMRI, and propose our opinions. It is expected that the analysis can supply several ideas for the researchers in Pre-AD diagnosis field.

## The analysis of Pre-AD based on sMRI

### Changes of brain structure and network via sMRI

Many studies related to the biomarkers of sMRI suggested that the atrophy of partial cerebral regions was regarded as a visual display of Pre-AD, which can use to predict or diagnose Pre-AD. Michael I. Miller et al. found that entorhinal cortex was the best sensitive brain region and also brain structure which changed at the earliest stages during the process of AD, and had a maximal atrophic rate in comparison with hippocampus and amygdala (Miller et al., 2015). Other studies also displayed that atrophic and mutation existed in entorhinal cortex at first (Miller et al., 2013; Younes et al., 2014), and the change was earlier than clinical manifestation about 8–10 years (Younes et al., 2014; Miller et al., 2015). When the change points across network were processed in the way of signal processing, different atrophic rate of intermediate caudal subarea of the entorhinal cortex would affect their own progress of signal processing (Miller et al., 2015). This result suggested that the nerve degeneration in Pre-AD presented a feature of selectivity.

Other relevant studies also suggested that variation of hippocampus and related brain regions could be regarded as the biomarkers. In 2015, Michael I. Miller et al. found that the hippocampus and amygdala atrophy appeared even later than entorhinal cortex, and the variation of amygdala appeared in basolateral and lateral areas of amygdala firstly (Miller et al., 2015). In addition, Laurent Younes et al. also found that the hippocampus atrophy happened in about 2–4 years before the clinical manifestation, and the amygdala atrophy also was earlier than clinical manifestation about 3 years (Younes et al., 2014). The study of Florent L Besson et al. suggested that volumes of hippocampus, ventro-medial prefrontal, fronto-insular, and lateral temporal cortex were less in sMRI-positive health elder (HE) than sMRI-negative HE (Besson et al., 2015). Anja Soldan et al. found that occurrence of the cognitive degeneration of Pre-AD can be predicted by the significant variation on standard volumes of right hippocampus and standard thickness of right and bilateral entorhinal cortex, and for example, the decrease of volume or thickness was related to the advanced clinical symptom (Soldan et al., 2015).

In addition, the change of brain networks also an important biomarker. Michael I. Miller et al. found that there existed abnormal change on substructure junction of medial temporal lobe (MTL) network of Pre-AD by analyzing high-field 11T atlasing sMRI (Miller et al., 2015).

### Changes of relationship between brain imaging via sMRI and protein, cognitive reserve, and gene

A few studies used multiple biomarkers including of sMRI to detect Pre-AD, and compared the performances of different biomarkers. Protein is the first biomarker related to brain imaging via sMRI. Florent L Besson et al. found that the volumes and metabolism velocity from AD-characteristic regions and prefrontal cortex of MRI-positive HE were decreased (Besson et al., 2015). The study also displayed that Aβ deposition was related to the appearance of Pre-AD symptom, and the insular, frontal, and temporal areas of HE with sMRI-negative had more Aβ deposition than HE with sMRI-positive (Besson et al., 2015).

Another biomarker is cognitive reserve (CR), which changed the relationship with brain imaging via sMRI when symptom of Pre-AD emerge. At the beginning of clinical symptom, CR values, and sMRI measures of hippocampus and amygdala volumes were not relative significantly (Soldan et al., 2015). Accounting for CR and ApoE genotype, the symptom of cognitive degeneration would appear earlier when right entorhinal cortex thickness, right and bilateral hippocampus volumes were smaller, which is consistent with the simple baseline models (Soldan et al., 2015). CR scores and baseline sMRI measures were significantly correlated on bilateral and left volume of entorhinal cortex (Soldan et al., 2015).

In addtion, gene is the last biomarker, which changed the relationship with brain imaging. Anja Soldan et al. found that only atrophy rate of left amygdala in the simple Cox regression models was related to ApoE-E4 genotype, which was regarded as a biomarker, when the clinical symptom was predicted (Soldan et al., 2015). Furthermore, there existed a larger atrophy rate in left amydala of ApoE-E4 genotype carrier relative to non-carrier, which was related to the earlier emergence of clinical symptom, and the relationship was not obvious on non-carrier (Soldan et al., 2015).

### Problems to be solved in the future

In the future, there exist three problems to be solved preferentially. From a localization perspective, future sMRI studies require to localize focal zones of Pre-AD precisely, which contribute to diagnosis, interposition, and treatment of Pre-AD. From a gene perspective, the correlation between Aβ deposition and brain volumes of sMRI-negative or sMRI-positive, also the relationship between ApoE-E4 genotype and the atrophy of specific brain regions all need to be quantified, in order to help us to directly diagnose Pre-AD via sMRI. From a CR perspective, we need to explore the correlation between other cognitive measures replacing CR and sMRI measures of left and bilateral volume of entorhinal cortex, so as to verify the sensibility of these brain regions in diagnosis of Pre-AD.

## The analysis of Pre-AD based on fMRI

### Changes of brain regions and functional connection via fMRI

A few studies in biomarkers suggested that there existed abnormities on function of brain regions and functional connectivity between different brain regions of Pre-AD (Sheline et al., 2010; Gordon et al., 2015; Harrison et al., 2016). Brian A.Gordon et al. expressed the deactivations and activations of Pre-AD brain regions by computing blood oxygen level via fMRI, and detected the change of attentional networks triggered by the task (Gordon et al., 2015). When the frequency of each word increased, precuneus presented smaller deactivation. And the activations of left dorsolateral prefrontal cortex increased obviously when the frequency decreased (Gordon et al., 2015). According to resting state fMRI study, Yvette I. Sheline found that for the non-cognitive impairment elders with and without amyloid plaques, the functional connectivity between precuneus and hippocampus, parahippocampus, visual cortex, superior precuneus, anterior cingulate, dorsal cingulate, gyrus rectus had significant differences (Sheline et al., 2010). The increase of amyloid plaques will affect the resting state functional connectivity of the subjects with normal cognitive before any abnormal cognition or behavior appear (Sheline et al., 2010). The influence mechanism of amyloid plaques to resting state functional connectivity contributes to the diagnosis of Pre-AD. In addition, the fMRI study of Theresa M. Harrison et al. also suggested that altered functional connectivity of hippocampus was regarded as an important biomarker of Pre-AD (Harrison et al., 2016).

### Alteration of brain network via fMRI

With the conversion from normal aging to AD, the functional networks consisting of different brain regions also are changed. Therefore, it is valuable to diagnosis of Pre-AD by quantifying functional networks. Matthew R. Brier et al. introduced graph theory to detection in change of brain network, which were regarded as a biomarker (Brier et al., 2014). In addition, they verified the accuracy of graph theory used to diagnose Pre-AD by studying the change of cerebrospinal fluid. The results showed that clustering coefficient decreased and modularity measure reduced significantly during cognitive decline, and suggested that large-scale broken connection had existed before the clinical symptom of Pre-AD appeared (Brier et al., 2014). With the increasing degeneration of cognitive function, Matthew R. Brier et al. observed that three important hubs on medial prefrontal cortices and anterior cingulate lost their own functions gradually from the phase of CDR 0 to CDR 1, and Pre-AD lay on the first phase of cognitive deterioration (Brier et al., 2014). In addition, a few researchers found that medial prefrontal cortex, bilateral lateral temporal cortex, bilateral parietal/angular cortex, posterior cingulated cortex/precuneus of normal control also played the role of hubs (Sepulcre et al., 2010; Drzezga et al., 2011).

### Relationship between brain imaging via fMRI and gene

A few studies suggested that clusterin genotype could affect the task-based and resting state fMRI paradigms of Pre-AD. The study of Green AE et al. showed that clusterin genetic risk and BOLD signals from MTL during an executive attention task were negatively correlated (Green et al., 2014). Erk S et al. found that healthy elder with clusterin had lower coupling strength between hippocampus and prefrontal cortex during memory retrieval tasks relative to healthy elder without clusterin (Erk et al., 2011). These studies implied that there existed modulatory relationship between BOLD signals and clusterin genotype from Pre-AD patients (Harrison and Bookheimer, 2016).

In addition, gene also affect functional connectivity between different brain regions of Pre-AD. The study of Theresa M. Harrison et al. displayed that healthy elder with ApoE-E4 gene had lower functional connectivity between anterior hippocampus and right precuneus, cingulate cortex, anterior insula, also between anterior hippocampus and posterior hippocampus with auditory cortex, right precuneus, bilateral supramarginal gyrus via fMRI on the retrieval phase of paired-associates memory task (Harrison et al., 2016). Reduction of functional connectivity between these brain regions can be an important biomarker of Pre-AD, suggesting that healthy elder with ApoE-E4 gene had a great risk suffering from Pre-AD.

### Problems to be solved in the future

In the future, there still exist three problems to be in urgent need of solution. Firstly, the functional connectivity need to be quantified, in order to evaluate Pre-AD accurately. In addition, the analysis methods of functional connectivity via sMRI and fMRI in the field of MCI require be improved, so that the improved methods are used to diagnosis of Pre-AD effectively. Secondly, complex network analysis methods also need be explored in the future, in order to distinguish Pre-AD from normal control more accurately at the level of brain network. Thirdly, considering that the studies about Pre-AD via fMRI can contribute to locate and find many specific genes, we need to build the corresponding relation between functional brain regions and specific genes on the brain model of monkey, and introduce the relationship to clinical diagnosis of Pre-AD.

## Conclusion

In conclusion, this paper analyzed in depth recent literatures related to analysis and diagnosis of Pre-AD from the angle of biomarkers via sMRI and fMRI. Although current studies obtained preliminary results, there still existed many problems to be solved in the future. Especially, it is a valuable angle that expand current studies from the level of analysis technology.

## Author contributions

YZ designed the study and wrote this paper, CT analyzed literatures, DW designed the study and wrote this paper, HS analyzed literatures, WH and YX revised this paper.

### Conflict of interest statement

The authors declare that the research was conducted in the absence of any commercial or financial relationships that could be construed as a potential conflict of interest.
